# Posterior cortical atrophy phenotypic heterogeneity revealed by decoding ^18^F-FDG-PET

**DOI:** 10.1093/braincomms/fcab182

**Published:** 2021-08-19

**Authors:** Ryan A Townley, Hugo Botha, Jonathan Graff-Radford, Jennifer Whitwell, Bradley F Boeve, Mary M Machulda, Julie A Fields, Daniel A Drubach, Rodolfo Savica, Ronald C Petersen, Matthew L Senjem, David S Knopman, Val J Lowe, Clifford R Jack, Keith A Josephs, David T Jones

**Affiliations:** 1 Department of Neurology, University of Kansas Medical Center, Kansas City, KS 66160, USA; 2 Department of Neurology, Mayo Clinic, Rochester, MN 55905, USA; 3 Department of Diagnostic Radiology, Mayo Clinic, Rochester, MN 55905, USA; 4 Department of Psychiatry and Psychology, Mayo Clinic, Rochester, MN 55902, USA; 5 Information Technology Radiology, Mayo Clinic, Rochester, MN 55905, USA

**Keywords:** posterior cortical atrophy, early-onset Alzheimer's disease, eigenvectors, FDG-PET, tau PET

## Abstract

Posterior cortical atrophy is a neurodegenerative syndrome with a heterogeneous clinical presentation due to variable involvement of the left, right, dorsal and ventral parts of the visual system, as well as inconsistent involvement of other cognitive domains and systems. ^18^F-fluorodeoxyglucose (FDG)-PET is a sensitive marker for regional brain damage or dysfunction, capable of capturing the pattern of neurodegeneration at the single-participant level. We aimed to leverage these inter-individual differences on FDG-PET imaging to better understand the associations of heterogeneity of posterior cortical atrophy. We identified 91 posterior cortical atrophy participants with FDG-PET data and abstracted demographic, neurologic, neuropsychological and Alzheimer’s disease biomarker data. The mean age at reported symptom onset was 59.3 (range: 45–72 years old), with an average disease duration of 4.2 years prior to FDG-PET scan, and a mean education of 15.0 years. Females were more common than males at 1.6:1. After standard preprocessing steps, the FDG-PET scans for the cohort were entered into an unsupervised machine learning algorithm which first creates a high-dimensional space of inter-individual covariance before performing an eigen-decomposition to arrive at a low-dimensional representation. Participant values (‘eigenbrains’ or latent vectors which represent principle axes of inter-individual variation) were then compared to the clinical and biomarker data. Eight eigenbrains explained over 50% of the inter-individual differences in FDG-PET uptake with left (eigenbrain 1) and right (eigenbrain 2) hemispheric lateralization representing 24% of the variance. Furthermore, eigenbrain-loads mapped onto clinical and neuropsychological data (i.e. aphasia, apraxia and global cognition were associated with the left hemispheric eigenbrain 1 and environmental agnosia and apperceptive prosopagnosia were associated with the right hemispheric eigenbrain 2), suggesting that they captured important axes of normal and abnormal brain function. We used *NeuroSynth* to characterize the eigenbrains through topic-based decoding, which supported the idea that the eigenbrains map onto a diverse set of cognitive functions. These eigenbrains captured important biological and pathophysiologic data (i.e. limbic predominant eigenbrain 4 patterns being associated with older age of onset compared to frontoparietal eigenbrain 7 patterns being associated with younger age of onset), suggesting that approaches that focus on inter-individual differences may be important to better understand the variability observed within a neurodegenerative syndrome like posterior cortical atrophy.

## Teaser

Townley et al. report that ^18^F-fluorodeoxyglucose PET combined with a novel decoding imaging analysis can capture the heterogenous presentation of posterior cortical atrophy. Left and right hemispheric differences accounted for 60% of the variance in this large cohort and were associated with demographic factors and clinical signs/symptoms.

## Graphical Abstract

**Graphical Abstract:**
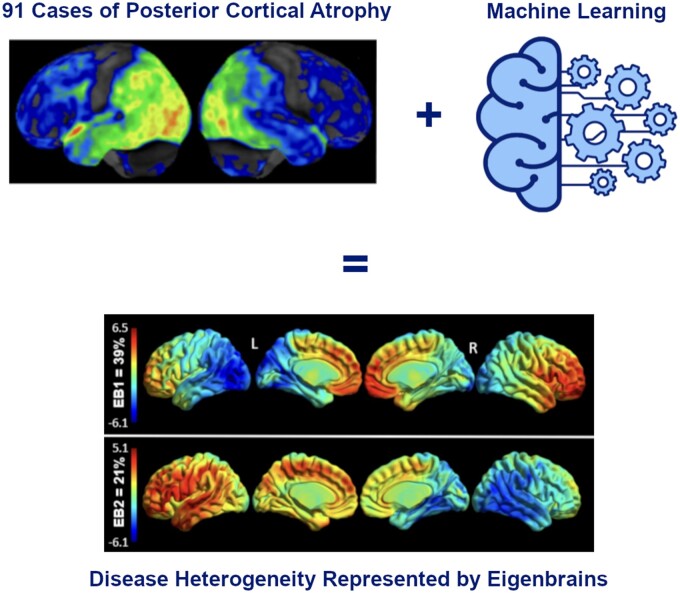

## Introduction

Posterior cortical atrophy (PCA) is a distinct neurodegenerative syndrome typically caused by Alzheimer’s disease pathology.^1^^,^^2^ Clinical diagnosis of posterior cortical atrophy is differentiated from typical amnestic Alzheimer’s dementia by visual dysfunction, younger age of onset and variable disease progression.^3^ PCA was first described in 1988^4^ but consensus criteria were not published until 2017.^5^ Ventral and dorsal visual streams of the left, right or bilateral hemispheres can be affected in posterior cortical atrophy, resulting in a wide variety of clinical symptoms. Recognizing and categorizing the phenotypic heterogeneity in PCA will help the field improve delays in diagnosis with early detection of PCA variants and may help us answer questions about long-term prognosis of these variants for patients and their families.

This heterogeneity has complicated the study of PCA,^6^ including neuroimaging studies, which often occur at the group level and focus on the group level correlates of symptoms or signs.^7–10^ Specifically, most neuroimaging studies have used clinical data or characterization as a starting point and then employed a mass-univariate regression framework to determine how the clinical data are ‘encoded’ in the imaging data (forward inference). This approach likely contributes to the seemingly inconsistent imaging findings often reported in degenerative diseases as it is not only sensitive to the specific cohort being studied, but also the particularities of the cognitive testing data used in the regression.

In recent years, the popularity of machine learning and latent variable analysis platforms has led to an increase in ‘decoding’ approaches.^11–14^ In this paradigm, the high-dimensional imaging data are used as the starting point, which is then used to derive a lower-dimensional representation or set of latent variables. The inference is then done using these lower-dimensional ‘patterns’ by predicting demographic or clinical data (reverse inference). The benefit of this approach is that the latent variables are based only on the imaging data and capture the rich large-scale biological variation in the cohort.

A data-driven study by Groot et al.^15^ used Bayesian modelling to identify latent atrophy factors in a large PCA cohort to assess associations between MRI atrophy patterns and cognition. In their analysis, some cognitive variables were associated with distinct atrophy patterns but the majority of individuals expressed multiple atrophy patterns arguing against straightforward classification into phenotypical variants. ^18^F-fluorodeoxyglucose (FDG)-PET is a more sensitive marker for regional neuronal dysfunction and is used routinely in clinical and research settings as a neurodegeneration biomarker at the single-participant level.^16^ This makes it an appealing modality to use in ‘decoding’ frameworks as it captures inter-individual variation well. In this article, we used an unsupervised machine learning framework to identify latent factors that capture the inter-individual variance in FDG-PET uptake in a large cohort of PCA participants. We hypothesized that the latent variables would capture important axes of disease heterogeneity and map onto demographic, clinical and biomarker data.

## Materials and methods

### Participant selection

The design and implementation of this single-centre retrospective study met HIPAA guidelines and was approved by the Mayo Clinic Institutional Review Board. Participants and/or their proxies signed a research document upon their first clinical visit to have their data used in research. Informed consent was obtained for clinical studies (e.g. lumbar puncture and FDG-PET) and subsequent research studies (e.g. tau and amyloid PET). There was no ICD9 code for posterior cortical atrophy, so the Mayo Clinic electronic medical records database was searched using terms: ‘posterior cortical atrophy’, ‘Benson’s syndrome’, ‘visual variant Alzheimer’s disease’ or ‘biparietal Alzheimer’s disease’ from dates 1 January 1999 through 1 September 2016. An MRI and FDG-PET scan were required for inclusion. Out of 186 individuals, a thorough and complete chart review took place to ensure they met the 2017 consensus criteria for PCA^5^ and had proper neuroimaging available, resulting in 91 total participants (Supplementary Fig. 1). Of these 91 participants, 24 had been recruited into the Neurodegenerative Research Group (NRG) (PI’s Joseph and Whitwell), and 11 had been recruited into the Mayo Clinic Alzheimer’s Disease Research Center (ADRC) (PI Petersen).

### Variables

Over 125 variables, including demographics, physical and clinical exam findings, neuropsychological results, AD biomarker results and neuroimaging results were recorded via thorough chart review. All clinical notes documenting signs and symptoms that were analysed via retrospective chart review took place prior to the FDG-PET scan acquisition. Clinical features were coded as present or absent based on clinical documentation. Variables were not recorded if they were not clearly described as positive or negative in the clinical history or the neurologic exam portion of the participant’s chart. Gerstmann’s syndrome^17^ and Balint’s syndrome^18^ were recorded as positive if all criteria for each syndrome were met. If one aspect, e.g. finger agnosia was not documented as present or absent, then ‘not available’ was documented for Gerstmann’s syndrome in that participant. Occupational history was recorded from the clinical note or neuropsychologist note and classified via International Standard Classification of Occupations (ISCO).^19^

### Cognitive testing

Bedside cognitive screening was done with the Kokmen Short Test of Mental Status.^20^ Further detailed neuropsychological testing was performed if indicated clinically or if they were enrolled in either our ADRC or NRG studies. The neuropsychological battery for each participant included combinations of the following tests: Dementia Rating Scale-2 (DRS-2),^21^ Rey Auditory Verbal Learning Test (AVLT),^22^ Wechsler Memory Scale-Revised (WMS-R) or 3rd Edition (WMS-III) Logical Memory (LM) I and Visual Reproduction (VR) I,^23^^,^^24^ Wechsler Adult Intelligence Scale-Revised (WAIS-R) or 3rd Edition (WAIS-III)—Digit Span (DS) and Letter Number Sequencing (LNS) subtests,^25^^,^^26^ Rey-Osterrieth Complex Figure copy,^27^ Trail Making Test Part A and B (Trails A, Trails B),^28^^,^^29^ Stroop Test: Word-Reading, Color-Naming, and Interference trials,^30^ Boston Naming Test (BNT),^31^ Controlled Oral Word Association Test (COWAT)^32^ and Category Fluency.^33^ Raw scores were converted to age-adjusted standard scores. Mayo Older Americans Normative Studies (MOANS) were used for all tests.^33–37^ The youngest MOANS age bracket (56–60) was used to derive normative scores for participants younger than age 56. All MOANS and standard scores were converted to *z*-scores for data presentation.

### Neuroimaging

^18^F-FDG-PET images were acquired using a PET/CT scanner (GE Healthcare) operating in 3D mode. Participants were injected in a dimly lit room with ^18^F-FDG, and after a 30-min uptake period, an 8-min ^18^F-FDG scan was performed, which consisted of four 2-min dynamic frames following a low dose CT transmission scan. Standard acquisition and vendor reconstruction parameters were used. PET images were normalized to an older adult template space^38^ and then intensity normalized to the pons and spatially smoothed with a 6 mm full-width half-maximum Gaussian kernel.^39^ The smoothed and normalized PET images were processed using a novel, in-house machine learning framework (details below). To visualize participant level PET findings, ^18^F-FDG-PET scans were also processed using CortexID software (GE Medical). The activity in each participant's PET dataset was normalized to the pons and compared with an age-segmented normative database, yielding *z*-score 3D-stereotactic surface projection images.

Amyloid-PET imaging was done with Pittsburgh compound B, synthesized on-site with precursor purchased from ABX Biochemical Compounds. Tau-PET was carried out with flortaucipir (^18^F-AV-1451), synthesized on-site with precursor supplied by Avid Radiopharmaceuticals. Image processing methods have been described previously.^11^^,^^40^ Amyloid and tau PET images were scaled using a cerebellar crus grey matter ROI, resulting in the standard uptake value ratio (SUVR) images. Previously validated Meta ROIs were used to derive a single value summary measure of amyloid and tau uptake. A positive amyloid-PET and tau-PET SUVR cut-off were defined as >1.42 and >1.23, respectively.^11^

### Between-subject variability projection and reduction

Details of the Between-subject variability Projection and Reduction (BPR) methodology have been described previously.^41^ Broadly speaking, the goal of BPR is to find a biologically interpretable, low-dimensional latent space that parameterizes the processes driving the inter-individual variation. Briefly, the BPR framework involves three key steps. In the first, voxel-wise variance is centred and standardized prior to subject-wise centring allowing for a high-dimensional representation of inter-individual variability of interest, such as a participant-by-participant covariance matrix. In the second step, this high-dimensional matrix is reduced to a low-dimensional representation through a technique like Singular Value Decomposition (SVD). Similar to the computation of ‘eigenfaces^42^’ one can use the decomposition of this smaller participant-by-participant matrix to determine the eigenvectors for the voxel-by-voxel matrix. An additional benefit of this approach is that the resulting eigenvectors retain the dimensionality of the masked template PET space. As such, it is trivial to represent the eigenvectors as ‘brain images’, which are referred to as ‘eigenbrains’, making interpretability far easier. These eigenbrains form the axes of the latent coordinate space, each of which has an associated set of weights for each participant, which can then be used to project existing participants, new participants, or linked biological and demographic data into the latent space in the third and final key step. In our prior work defining BPR, we explored the impact of different participant populations, the choice of distance or similarity metric used, and several options for data reduction, among many validation steps. We do not repeat these here, but it is worth emphasizing that the latent space is naturally dependent on the population used to derive it. In our case, we would only aim to identify a small part of what has been termed the Global Functional State Space since we are only including participants with PCA. The first 8 eigenbrains (EB1-8) explained 50% of the variance and were used for further analyses.

### Neurosynth analysis

The NeuroSynth (www.neurosynth.org) online decoding tool was used to assign functional topic terms to each of the EBs. In essence, using a large database of neuroimaging studies, NeuroSynth provides the cognitive topics that best align with the positive and negative loadings in a template-space brain image. This can then be used to interpret the cognitive axes captured by a given EB, with reference to a large body of functional neuroimaging studies.^43^^,^^44^

### Statistical analysis

All statistical analyses were performed using R statistical software. A regression framework was used to determine the relationship between the eigenbrains and clinical data, with the clinical variable of interest as the dependent variable and participant loads on the 8 eigenbrains as independent variables. For continuous variables, linear regression was used and for binary variables, we used logistic regression. To simplify the comparison of regression coefficients across eigenbrains, the weights were centred (mean = 0) and scaled (SD = 1). The same scaling was applied to continuous dependent variables. This meant that, for a continuous dependent variable, the coefficients could be interpreted as the magnitude of change in the dependent variable, measured in SDs, for a single SD change in the independent variable. Similarly, for the binary dependent variables, the coefficients represented the change in the odds of the binary variable being true/present for a single SD change in the independent variable. Statistical significance was defined as *P* < 0.05 and the false discovery rate correction was applied to correct for multiple comparisons.

### Data availability

Data that support the findings in this study are available from the corresponding author upon reasonable request.

## Results

### Demographics

Mean years of age at clinical symptom onset was 59.3 with an average disease duration of 4.2 years prior to FDG-PET scan. Females were more common than males at 1.6:1 with an average education of 15.0 years (Table 1). The most recent participant occupation data were available in 83/91 participants (6 were listed as homemakers and 2 with unavailable information) and are described in Supplementary Table 1. Of the 10 major ISCO groups, 54 participants (65%) made up the highest skill level of occupations with 41 participants (49.4%) being professionals (17 were teachers ranging from elementary school to university professors), and an additional 13 participants (15.6%) being high-level executives and business owners. No statistical difference was found in EB weights across occupational skill levels.

**Table 1:** Demographics and clinical data

**Demographics (*n* = 91)**	

Gender, F—*n* (%)	56 (61.5)%
Age of onset, mean years (SD)	59.3 (7.0)
Symptom duration before scan, mean years (SD)	4.2 (1.9)
Education (years), mean (SD)	15.0 (2.9)

**AD biomarkers/APOE4 status**	

CSF only, *n* = 16	A+ (16), T+ (11), T− (5)
Amyloid PET only, *n* = 15	A+ (15)
CSF and Amyloid PET, *n* = 7	A+ (7), T+ (6), T− (1)
CSF and Amyloid and Tau PET, *n* = 5	A+ (5), T+ (5)
No CSF and Amyloid and Tau PET, *n* = 9	A+ (9), T+ (9)
Amyloid-PET SUVR, *n* = 36, (median IQR)	2.39 (2.18–2.69)
Tau-PET SUVR, *n* = 14, (median IQR)	2.25 (1.92–2.37)
CSF amyloid, *n* = 28, (median IQR)	302.4 (235.6–388.5)^a^
CSF total tau, *n* = 28, (median IQR)	494.9 (351.9–832.3)^a^
CSF p-tau, *n* = 28, (median IQR)	83.6 (57.8–103.9)^a^
Amyloid Tau Index, (median IQR)	0.33 (0.26–0.48)
*APOE* ε4 positivity	6/15

a Reported in pg/ml.

A, amyloid; APOE, apolipoprotein E; IQR, interquartile range; SD, standard deviation; SUVR, standardized uptake value ratio; T, phosphorylated tau.

### Biomarker confirmation

Of the 91 participants, 54 participants underwent AD biomarker evaluation and all 54 had testing consistent with AD pathophysiology. A combination of biomarker confirmation included: 36 amyloid-PET scans, 14 Tau-PET scans, 28 CSF biomarkers and 8 autopsy studies. The breakdown of amyloid (A), tau (T) positive or negative status for biomarkers and molecular PET scans, along with median and interquartile ranges (IQRs) for each biomarker are provided in Table 1. Of note, there were 6 total participants that were amyloid positive but tau negative on CSF, consistent with our experience with this commercial biomarker assay in young-onset Alzheimer’s disease variants.^45^

### Clinical variables

Each variable in the updated 2017 PCA consensus criteria is listed as a fraction of positive over total documented within Table 2. The most common positive clinical symptoms were reading difficulties, memory loss, object perception deficit, space perception deficit and environmental agnosia. The most common physical exam findings recorded at clinic presentation were constructional apraxia, acalculia and simultanagnosia.

**Table 2:** Clinical findings

Clinical and physical exam featuresa	
Object perception deficit	73/73 – (100%)
Space perception deficit	57/57 – (100%)
Constructional apraxia	83/85 – (97.6%)
Simultanagnosia	79/82 – (96.3%)
Reading difficulties	71/76 – (93.4%)
Acalculia	82/91 – (90.1%)
Memory loss	79/91 – (86.1%)
Environmental agnosia	52/62 – (83.6%)
Anxiety	28/35 – (80.0%)
Visual hemineglect	22/29 – (75.9%)
Dressing apraxia	32/44 – (72.7%)
Ideomotor apraxia	45/66 – (68.2%)
L/R disorientation	19/29 – (65.5%)
Agraphia	18/28 – (64.3%)
Ocular apraxia	33/54 – (61.1%)
Optic ataxia	34/58 – (58.6%)
Finger agnosia	13/23 – (56.5%)
Complete Balint’s syndrome	21/49 – (42.8%)
Myoclonic jerks	14/33 – (42.4%)
Aphasia	23/60 – (38.3%)
Prosopagnosia	17/48 – (35.4%)
Complete Gerstmann’s syndrome	8/29 – (27.6%)

a Frequency of presence/total number documented for each test.

### Cognitive test scores

A bedside cognitive screening test was performed in 83/91 participants with a median short test of mental status (STMS) of 26/38, which is equivalent to a Montreal Cognitive Assessment score of 16/30.^46^ Points were most commonly missed on subtests of calculation, construction and delayed memory (which comprises 12 points of the STMS). A profound visual difficulty was the primary reason listed for not performing a complete bedside cognitive screening in the other 8 participants. Additionally, 55/91 participants underwent a portion of detailed neuropsychological testing. Immediate verbal memory tests (AVLT, Logical Memory I) were less affected compared to visuospatial related tasks (Visual Reproduction I, Block Design, Picture Completion, Spatial Span, Digit Symbol, Trails A/B, Rey-O) which were consistently most affected with *z*-scores listed in the Neuropsychological Results portion of Table 3. Language-based testing showed minimal impairment on the BNT and COWAT and moderate impairment on Category Fluency. Trails B had a significant floor effect as 39/55 could not complete the test in the maximum time allowed and was coded as 300 s (MOANS of 1 ∼ *z*-score of −3). Trails A also had 19/55 that could not complete the task and were coded as a *z*-score of −3. Executive functioning tests revealed minimal difficulty on non-visual based tasks (Digit Span) compared to Stroop testing, which was likely affected by visual difficulties (all three subtests on Stroop had the same *z*-score).

**Table 3:** Cognitive testing

Neuropsychological results	
STMS, Median (IQR)	26 (21–30)
Calculation score (mean out of 4)	1.34
Construction score (mean out of 4)	0.95
Dementia Rating Scale 2, *n* = 37	−1.7^a^
Digit Span, *n* = 44	−0.3^a^
Letter Number Sequence, *n* = 36	−1.0^a^
Picture Completion, *n* = 29	−1.8^a^
Block Design, *n* = 41	−2.0^a^
Matrix Reasoning, *n* = 36	−1.3^a^
Digit Symbol, *n* = 21	−2.0^a^
Spatial Span, *n* = 22	−2.0^a^
WMS III Logical Memory I, *n* = 52	−1.3^a^
WMS III Visual Reproduction I, *n* = 46	−2.2^a^
AVLT Trial 1, *n* = 55	−0.8^a^
AVLT Delayed Recall, *n* = 55	−1.2^a^
Trails Making Test A, *n* = 55	−2.3^a^
Trails Making Test B, *n* = 55	−2.6^a^
Stroop Word, *n* = 19	−2.1^a^
Stroop Color, n = 19	−2.1^a^
Stroop Interference, *n* = 19	−2.1^a^
COWAT, *n* = 48	−0.3^a^
Category Fluency, *n* = 49	−1.2^a^
BNT, *n* = 36	−0.7^a^
Rey-O, *n* = 46	−2.5^a^

a *Z*-score.

AVLT, Auditory Verbal Learning Test; BNT, Boston Naming Test; COWAT, Controlled Oral Word Association Test; IQR, Interquartile Range; Rey-O, Rey-Osterrieth complex figure; STMS, Short Test of Mental Status (score out of 38); WMS, Wechsler Memory Scale

### Eigenbrains

**Figure 1:**
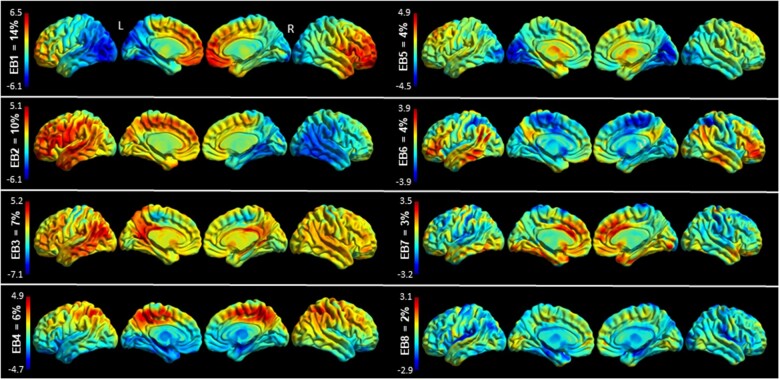
**Eigenbrains Surface renderings of the eight eigenbrains that explain over 50% of the variance of this sample.** The percentage of cumulative variance explained by each eigenbrain is displayed above each colour bar. The colour bar encodes positive (red) and negative (blue) intensities for each eigenbrain. While a given participants’ scan can be approximated by a weighted linear combination of the above eigenbrains, the above images are not direct representations of hypometabolism or preserved metabolism. Rather, positive (red) regions indicate *relatively* preserved metabolism, and negative (blue) regions indicate *relatively* reduced metabolism. Furthermore, the signs of all the eigenbrains are indeterminate—if all the eigenbrain signs and participant weight signs are flipped about zero the decomposition would be equally valid. Inference occurs at the pattern—or eigenbrain—level, using the participant weights on the entire pattern.

Eight eigenbrains accounted for over 50% of the cumulative variance in this cohort and are shown in Fig. 1.

**Figure 2:**
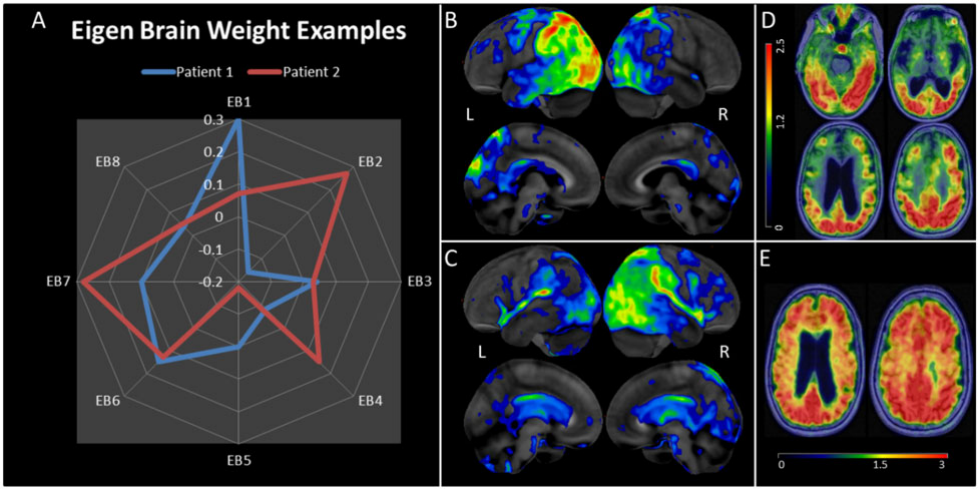
**Participant level examples of the relationship between eigenbrain weights and participant PET data.** (**A**) Eigenbrain weight distributions (EB1–EB8 shown in Fig. 1) for two individuals. (**B**) Patient 1 ^18^F-FDG-PET (Cortex ID from GE Healthcare, *z*-score is 0 to −7): a 63-year-old female presented with a 7-year history of visual predominant symptoms. She went to multiple eye doctors, tried numerous eyeglass prescriptions, and underwent an operation for cataracts which did not improve her visual problems. Within two years she developed significant apraxia of her right hand, being unable to shift gears in her car, difficulties writing, and difficulties opening jars. She developed aphasia after 4 years. By the time she presented to the clinic, she was not testable on STMS due to severe aphasia. She had prominent optic apraxia, myoclonic jerks and severe right upper extremity apraxia. CSF AD biomarkers were positive (A+/T+). The FDG-PET revealed left-sided asymmetric hypometabolism and was consistent with the EB1 positive weighting and EB2 negative weighting. The presence of aphasia and ideomotor apraxia was consistent with EB1 odds ratios as well. (**C**) Patient 2 ^18^F-FDG-PET: a 66-year-old female presented with a 5-year history of progressive visual predominant symptoms. Her first symptom was described as visual blurriness which did not improve with LASIK surgery. This progressed to exhibiting inattentiveness and getting lost in familiar locations. Two years after symptoms she developed left visual hemifield neglect along with left upper extremity apraxia and myoclonic jerks. She scored 32/38 on STMS, 0/4 points on construction and 2/4 points on delayed recall. She had optic apraxia, simultanagnosia, left upper extremity apraxia, and astereognosis of the left hand. She was enrolled in a Mayo Clinic neuroimaging study and had a positive amyloid and tau PET scan. (**D**) Patient 2 ^18^F-AV1451 tau PET axial slices (SUVR range to the left—mean SUVR 1.64). (**E**) Patient 2 PiB amyloid PET (SUVR range at the bottom—mean SUVR 2.48).

All eight eigenbrains explained over 50% of the variance in age of onset, DRS-2 total score, Trails A score and Block Design (Table 4). EB1 captured negative weighting in left hemispheric ventral and dorsal visual streams with positive weights towards the right > left frontal lobes. EB2 nearly mirrors EB1 with negative weighting in right hemispheric ventral and dorsal visual streams with positive weights largely overlapping language networks in the left > right frontal and temporal lobes. Together, these two important EBs account for 24% of the variance in this cohort. Figure 2 portrays two participants weighted heavily to these two important EBs and shows their individual FDG-PET scans for comparison. Note that other EB weights vary between the two individuals, and cumulatively, these eigenbrains explain the glucose uptake patterns for each individual, but individually the EBs are not a direct correlation of hypometabolism severity.

**Table 4:** Continuous and binary variables compared across eigenbrains

Continuous variables beta coefficients (bold = significant)																	
Variable	*R* ^2^	_Adj_ *R* ^2^	*N*		EB1		EB2	EB3	EB4	EB5	EB6	EB7	EB8	*P*-value			*P*-value FDR
Age of onset^a^	0.52	0.47	91		−0.14		−0.02	**0.21**	**0.48**	0.01	**0.21**	−**0.37**	**0.18**	**<0.001**			**<0.001**
Education	0.14	0.05	91		−0.01		−0.17	**0.23**	0.13	0.17	0.07	0.06	−0.04	0.13			0.94
STMS	0.41	0.35	83		−**0.39**		0.12	**0.39**	0.06	0.14	0.17	**0.28**	−**0.25**	**<0.001**			**<0.001**
Calculation	0.40	0.33	83		−0.15		**0.20**	**0.25**	**0.24**	**0.20**	**0.21**	**0.22**	−**0.31**	**<0.001**			**<0.001**
Construction	0.27	0.19	81		−**0.20**		−**0.21**	**0.23**	**0.23**	**0.20**	0.13	0.13	−**0.20**	**<0.01**			**<0.05**
DRS-2 Memory	0.43	0.27	39		−**0.40**		−**0.31**	**0.39**	−0.01	0.01	0.06	**0.35**	−**0.29**	**<0.05**			0.33
DRS-2 Total	0.57	0.46	39		−**0.31**		−**0.27**	**0.58**	0.10	0.11	−0.01	**0.45**	−**0.38**	**<0.001**			**<0.05**
Block design	0.52	0.39	42		−**0.37**		−**0.48**	**0.51**	0.21	−0.03	−0.04	−0.07	−0.15	**<0.01**			**<0.05**
Visual Rep. I	0.40	0.27	46		−0.14		−0.21	**0.61**	0.20	0.03	−0.13	0.16	−0.23	**<0.01**			**0.15**
Rey-O	0.38	0.26	51		−**0.47**		−**0.25**	**0.48**	0.21	−0.06	−0.10	−0.02	−0.17	**<0.01**			0.10
Trails A	0.53	0.45	55		−**0.52**		−0.10	**0.62**	**0.27**	−0.19	−0.04	**0.27**	−**0.22**	**<0.001**			**<0.001**
Trails B	0.38	0.28	55		−**0.35**		−0.11	**0.47**	**0.38**	−0.07	0.08	0.11	−0.10	**<0.01**			**<0.05**
COWAT	0.31	0.17	48		−0.23		−0.11	**0.38**	0.07	0.17	0.01	**0.34**	−**0.35**	**<0.05**			0.40
Category Fluency	0.42	0.31	50		−0.21		0.02	**0.56**	−0.11	0.19	0.20	0.25	−**0.32**	**<0.01**			**<0.05**
BNT	0.34	0.16	38		−0.14		−**0.32**	**0.47**	−0.05	−0.08	0.15	0.23	−**0.39**	0.10			0.91

**Binary variables odds ratio** (**bold = significant)**																	

**Variable**				***N***		**EB1**	**EB2**	**EB3**	**EB4**	**EB5**	**EB6**	**EB7**	**EB8**		***P*-value**	***P*-value FDR**	

Sex^b^				91		1.03	1.48	**0.50**	0.92	0.91	0.66	1.02	0.74		0.06	0.14	
Environmental agnosia				62		2.15	**2.88**	0.67	0.55	0.76	0.99	1.30	1.06		0.11	0.14	
Apperceptive prosopagnosia				48		1.30	**2.45**	1.22	2.07	1.11	0.57	0.44	1.71		**<0.05**	0.14	
Aphasia				60		**2.94**	**0.47**	**0.22**	0.95	0.58	0.50	1.02	1.61		<**0.001**	**<0.01**	
Apraxia				66		**2.14**	0.96	**0.41**	**0.35**	**0.50**	1.20	1.45	1.51		**<0.05**	**<0.05**	
Memory loss				91		**4.48**	1.67	**0.11**	**3.16**	**0.21**	0.50	1.53	**3.32**		**<0.05**	**<0.01**	
Myoclonic jerks				33		1.36	1.99	**0.10**	1.54	0.80	**2.97**	2.07	**9.60**		**<0.001**	**<0.01**	

a Negative number indicates younger age, and positive number indicates older age.

b Odds ratio for male.

BNT, Boston naming test; COWAT, controlled word association test; DRS-2, dementia rating scale 2; EB, eigenbrain; FDR, false discovery rate; Rey-O, Rey-Osterrieth complex figure; STMS, short test of mental status.

As in the ‘Patient 1’ example in Fig. 2, participants weighting heavily to the left-sided EB1 pattern were significantly more likely to have aphasia (OR 2.94), memory problems (OR 4.48), ideomotor apraxia (OR 2.14), and difficulties on the STMS (*β* = −0.39) and global cognitive decline measured by multi-domain neuropsychological testing difficulties (Table 4).

**Figure 3:**
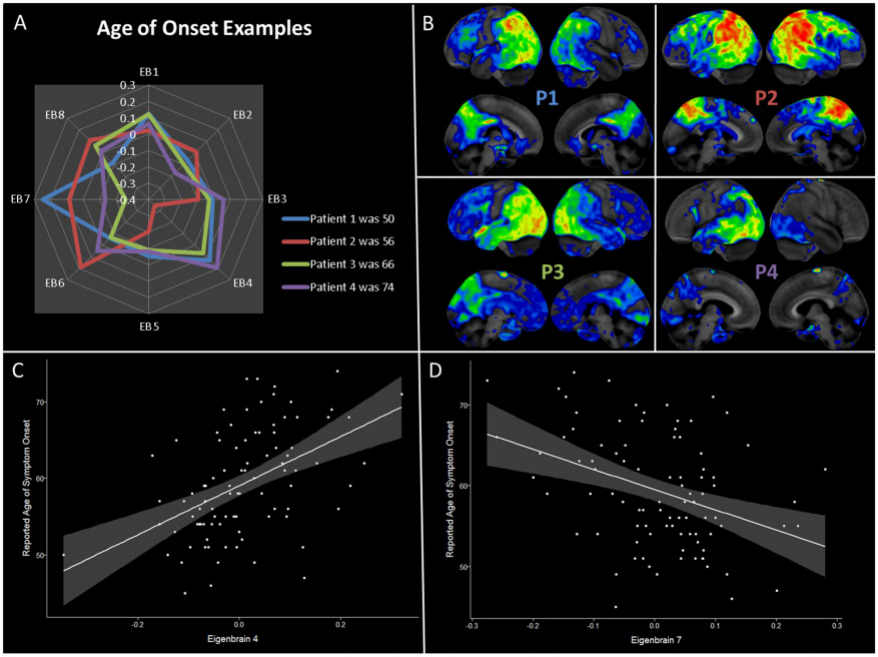
**Relationship between age of onset and eigenbrain weights.** Age of reported symptom onset was significant across multiple eigenbrain vectors (with over 50% of variance explained by eigenbrain vectors), but this association was strongest in EB4 and EB7. (**A**) Four individuals with a wide variation in age are mapped onto the eight primary eigenbrains. Positive weighting to EB7 and/or negative weighting to EB4 are associated with younger age of symptom onset (Patient 1 and 2). Positive weighting to EB4 and/or negative weighting to EB7 are associated with older age of symptom onset (Patient 3 and 4). (**B**) The four individual FDG PET scans are shown in the same anatomical orientation as Fig. 2. (**C**) Eigenbrain 4 has a significant association with reported age of symptom onset (*r*^2^ = 0.23, *t*-value 5.2, *P* < 0.001). (**D**) Eigenbrain 7 had an opposite but significant association with reported age of symptom onset (*r*^2^ = 0.15, *t*-value −3.8, *P* < 0.001).

Participants weighting heavily to the right-sided EB2 pattern were less likely to have symptoms of aphasia (OR 0.47) and less severe calculation problems (*β* = 0.20) but were more likely to have environmental agnosia (OR 2.88), apperceptive prosopagnosia (OR 2.45), and struggled with DRS-2 construction, DRS-2 memory, and block design on detailed testing. EB3 accounted for 7% of the variance and captured positive weighting in language networks involving the left > right temporal and inferior parietal lobe. The posterior cingulate and precuneus also had positive weighting in EB3. Participants who weighted heavily towards EB3 had higher education levels (*β* = 0.23), were less likely to be male (OR 0.50), and had an older age of onset (*β* = 0.21). They had significantly higher neuropsychological scores (increased *β* coefficients) and fewer clinical findings of aphasia, apraxia, myoclonic jerks or memory loss (significantly decreased ORs). EB4 accounted for 6% of the variance and captured a limbic predominant brain network with negative weighting to the medial and anterior temporal lobes and positive weighting to the precuneus and superior parietal cortices. Participants weighting heavily towards EB4 had a significantly older age of onset (*β* = 0.48) and more frequency of memory loss (OR 3.16), but less frequency of apraxia (OR 0.35), fewer calculation difficulties (*β* = 0.24), less difficulty with Trails A/B (*β* = 0.27/0.38), and trended towards less simultanagnosia (not shown in Table 3) but simultanagnosia did not meet statistical significance (*P* = 0.10). EB5 accounted for 4% of the variance and showed negative weighting in primary visual and medial occipital lobes. Positive weighting towards medial temporal and frontal lobes were also captured in EB5. Participants weighting towards EB5 had fewer memory problems (OR 0.21), less apraxia frequency (OR 0.50), and less calculation or construction difficulties (*β* = 0.20). EB6 accounted for 4% of the variance and positive weights overlap well with the default mode network, including the precuneus, posterior cingulate, angular gyrus, lateral temporal, and lateral and medial prefrontal cortex. Negative weights were captured in the superior parietal lobes and the medial sensorimotor cortex. Participants weighting towards this pattern had older age at onset (*β* = 0.21), were more likely to have myoclonic jerks (OR 2.97), and had less calculation difficulties (*β* = 0.21). EB7 accounted for 3% of the variance and the positive and negative weights were nearly inverted from EB4 with positive weights along the limbic network involving the medial and anterior tempora lobes and the anterior cingulate with mild negative weights in the precuneus and parietal cortices. There was a strong association between younger age of onset and higher loads on EB7 (*β* = −0.37) and participants with this pattern also did better across most neuropsychological tests. The opposing direction of association in the reported age of symptom onset between EB4 and EB7 are shown in Fig. 3. EB8 accounted for 2% of variance and there is negative weighting towards the limbic networks, sensorimotor cortrex and the sylvian fissures. Participants weighting towards EB8 had an older age of onset, were more likely to have myoclonic jerks (OR 9.60), and did worse on most neuropsychological tests.

Results for the *Neurosynth* based eigenbrain decoding are shown in Supplementary Table 2. The main positive and negative associations, respectively for each eigenbrain, were consistent with the behavioural data in our cohort, such as language (positive) and visuospatial function and facial recognition (negative) for eigenbrain 2, or executive function/attention (positive) and memory and conditioning (negative) for eigenbrain 4.

## Discussion

In this study, we present an analysis of a large cohort of PCA participants with heterogeneous patterns on metabolic imaging that informs the biological factors related to this clinical dementia syndrome. The factors that drive heterogeneous presentations in posterior cortical atrophy are not well understood. Individuals can present with stark asymmetry, bilateral disease, or variations in dorsal or ventral visual stream involvement. We used a novel analysis technique, Between-subject variability Projection, and Reduction, to identify eight ‘eigenbrains’, latent vectors that represent the principal axes of inter-individual variation in a lower-dimensional space. In this framework, PCA can be thought of as a disease process that is parameterized by a set of eigenbrains, each of which maps onto important clinical data, and an individual PCA participant’s PET scan can be represented as a linear combination of these eigenbrains. Furthermore, we showed that these eigenbrains mapped onto demographic factors, such as age of onset, neurologic and neuropsychological data in our sample, as well as cognitive functions from the *Neurosynth* online database. This suggests that a data-driven ‘decoding’ approach can capture important, disease-relevant biological patterns in a heterogeneous cohort. Taken together, the reverse inference or ‘decoding’ approach employed here allowed us to capture important biological information by focussing on inter-individual differences in disease expression. We will expand on the biological relevance of the eigenbrains by discussing their relationship to clinical data in our cohort as well as imaging findings from the broader literature.

### Left versus right asymmetry

The first two eigenbrains, which accounted for ∼24% of the variance, were highly lateralized, suggesting that this is a major source of heterogeneity in our cohort. More right-sided involvement (higher loads on EB2) was associated with apperceptive prosopagnosia (OR 2.45), environmental agnosia (OR 2.88), less aphasia (OR 0.47) and less mental calculation difficulties (*β* = 0.20). In contrast, more left-sided involvement (higher loads on EB1) was associated with worse Trails A/B performance, which is a test that requires both visual processing and executive functions of mental set shifting (more specific to Trails B). The difficulty completing this task could be related to a combination of dorsal visual pathway involvement and the dominant parietal lobe overlapping with the working memory network responsible for executive function.^47^ There was an increased frequency of aphasia documented in clinical notes (OR 2.94), but their difficulties on language-based neuropsychological tests did not reach statistical significance. We note that EB1 weighted participants did significantly worse on bedside cognitive testing (*β* = −0.39), and due to this severe impairment were less likely to undergo detailed neuropsychological testing that would have included detailed language tests. *Neurosynth* decoding results for EB1 and EB2 were consistent with these data: regions loading negatively (blue) on EB1 and positively (red) on EB2 were associated with language functions, whereas negatively loaded areas on EB2 were associated with face recognition and visual construction difficulties.

### Dorsal versus ventral visual pathways

Some of the identified eigenbrains, most notably EB4, appeared to capture differential involvement of the dorsal and ventral streams, the two primary higher-order visual processing pathways.^48^ The dorsal stream projects from the occipitoparietal cortex to the posterior parietal cortex before its path diverges anteriorly into the dorsolateral prefrontal cortex for spatial working memory and the premotor cortex for visually guided movements.^49^ The dorsal stream is also important for spatial navigation with projections medially through the posterior cingulate cortex and the retrosplenial cortex to the medial temporal lobe.^49^ The ventral stream projects from the occipitotemporal cortex to the anterior temporal lobe to the ventrolateral prefrontal cortex and is important for processing perceptual dimensions and features of visual information.^50^ These two main pathways also communicate via the vertical occipital fasciculus.^51^

Clinical testing of different visual processing pathways is a growing area of interest to improve PCA diagnostic accuracy. Ventral stream cognitive tests include face recognition, colour recognition and object naming.^52^ Apperceptive prosopagnosia, as tested with recognition of famous faces, was indeed associated with higher loads on EB4, supporting the idea that this eigenbrain captured ventral stream involvement.^53^ Colour recognition tests were rarely tested or formally recorded in our cohort and are not reported here. The Stroop subtests were abnormal at a group level (*z*-score = 2.01) and colour recognition could be part of this difficulty, but there were no statistical differences across EBs. Previous studies suggest ventral stream predominance is associated with greater confrontational naming impairment compared to dorsal stream predominance.^54^ However, we did not find an association between EB4 and the Boston Naming Test.

Dorsal stream cognitive tests typically include reaching for objects, assessing extraocular movements for apraxia, testing complex picture reproduction (Rey-O figure) and compound overlapping stimuli assessing simultanagnosia.^55^ Since EB4 appeared to capture positive dorsal stream areas, one would anticipate a negative association between EB4 and simultanagnosia. A quantitative score for simultanagnosia severity was not recorded in the participant’s chart, and either a present/absent was coded. As a binary variable, 79/82 had some form of simultanagnosia, but there was still a trend towards significance between EB4 and absent simultanagnosia (the 3 absent cases weighted heavily to EB4, *P* = 0.10). Future studies utilizing quantitative scoring of simultanagnosia severity could better analyse the difference between ventral or dorsal stream on this clinical measure.

Similar to Groot et al.,^15^ we did not find distinct phenotypes of right-ventral, right-dorsal, left-ventral or left-dorsal in the majority of cases. Looking at the two most important EBs defining this cohort, EB1 and EB2, there is an overlap with both the ventral and dorsal streams. The differentiating factor in this cohort’s disease stage appears more related to hemispheric lateralization than separation into ventral and dorsal streams. The average reported symptom onset was over 4 years prior to FDG-PET scan, this timepoint could be too far along in the disease process to separate out early changes in different visual streams. This delay to diagnosis/PET scan may also explain part of the low 4% variance of the caudal/occipital lobe predominant EB5 pattern. Studying earlier stages of the disease to assess whether there are discrete ventral and dorsal stream patterns would be needed to test that hypothesis, and FDG-PET would be a preferable imaging modality over MRI.^16^

### Demographics

The age of reported symptom onset varied greatly across our cohort (range 45–74) and the eight eigenbrain patterns explained over 50% of this variance. Multiple eigenbrain patterns had statistical significance in association with age of onset (EB3, 4, 6, 7 and 8). As shown in Fig. 3, EB4 and EB7 have the strongest and opposing associations with age and have opposite patterns of positive and negative weights for posterior cingulate, precuneus, parietal, lateral temporal, medial temporal, anterior temporal, anterior cingulate and orbitofrontal regions. Interestingly, EB4 has clear overlaps with the limbic network and may represent comorbid limbic TDP-43 pathology that is more common in older individuals.^56^^,^^57^ The large-scale organization of the brain, both within or across brain networks, changes with age,^58–60^ and insight into these changes may help predict normal and abnormal ageing. A recent paper showed that preserved FDG-PET uptake in the bilateral anterior cingulate and anterior temporal pole was associated with stable baseline global cognition in older individuals.^61^ The high intensity (red) regions of EB7 overlap with this resilience pattern of anterior cingulate and anterior temporal lobe involvement. It may be that anterior cingulate metabolism declines with age and is relatively maintained in resilient participants, which explains the negative association between age and EB7 as well as the positive association between EB7 and better cognitive performance seen in our cohort, perhaps indicating some form of cognitive resilience in the setting of early symptoms (Table 4). Additionally, participants weighting towards an EB3 pattern had preserved cognitive functions across all measured variables and these participants had higher education levels, a well theorized factor in cognitive resilience.^62^ The positive association between age and EBs 3, 4, and 6 suggests that older participants had relatively preserved frontoparietal metabolism. This is consistent with the early-onset Alzheimer’s disease literature, where younger patients have been shown to have more involvement of the frontoparietal regions, including MRI atrophy, FDG-PET hypometabolism and higher binding on tau PET.^45^^,^^63–65^ Taken together, this may suggest that patterns of frontoparietal involvement have an important association with younger participants, but anterior cingulate region compensation may help attenuate the severity of cognitive impairment.

The occupational history is similar to a previous report in Speech and Language disorders where teachers made up 22% of the cohort and non-teacher professionals were another 16%.^66^ In this cohort, 17/83 (20%) were teachers with an additional 37/83 (45%) being non-teacher professionals (i.e. physicians, therapists, accountants, programmers, engineers, business owners and high-level executives of corporations—Supplementary Table 1). There are potentially two different types of referral biases present. Troubles with reading was the most common first symptom noted across all participants, and higher skill occupations, particularly teaching, require higher volumes of reading. Thus, sensitivity to these early symptoms may be an impetus to seek evaluation sooner. However, the mean time to clinical diagnosis was often over 4 years in our participants, owing largely to difficulty in diagnosing early visual symptoms in PCA patients outside of specialty referral centres. Secondly, Mayo Clinic is a tertiary referral centre and may be more likely to see participants with higher socioeconomic status.^67^ The above-mentioned Josephs study was also performed at Mayo Clinic. Even considering these biases, it is difficult to dismiss the number of participants with higher skill jobs (71.2%) compared to lower-skill jobs (28.8%) as defined by the ISCO. Further epidemiology-based studies that involve community populations would be helpful to address the academic referral bias, but it may prove difficult to accurately diagnose PCA outside specialized academic centres for the time being. There was no association with occupational groups and specific eigenbrains, and we did not test eigenbrains against individual occupations due to low power.

Limitations of the present study include its retrospective nature and lack of standardized clinical assessments. For example, although we found that apraxia and anxiety were highly prevalent, these were not universally documented, raising the possibility that negative or subtle symptoms went undocumented. Similarly, several participants did not undergo neuropsychological testing, bringing up the possibility of selection bias and a non-representative sample for participants who had these tests performed (e.g. confrontational language testing discrepancy in aphasic participants). In future studies, it will be important to record these variables from the start of a study and in a consistent manner.

## Conclusions

PCA is a heterogeneous disorder and a novel decoding imaging analysis approach was able to capture these heterogeneous presentations. We used 2017 PCA criteria to describe detailed clinical symptoms, exam findings, Alzheimer’s biomarkers and neuropsychological data at a group level in a large series of participants. Furthermore, the inter-individual differences among these variables can be investigated with this novel imaging analysis. Similar to previous studies,^15^ discrete right-ventral, right-dorsal, left-ventral and left-dorsal phenotypes may prove difficult to subtype at later stages of the disease (over 4.2 years after reported symptom onset in our cohort). However, utilizing sensitive imaging tools like FDG-PET that are suitable for both research and clinic settings may help improve early detection and our ability to subtype PCA patients more accurately at earlier disease stages. In addition to improving our understanding of the heterogeneity within PCA, this method could be used for more diverse cohorts with FDG-PET data to investigate robust inter-individual differences to better understand the variability observed within and across neurodegenerative syndromes at large.

## Supplementary material

Supplementary material is available at *Brain Communications* online.

## Supplementary Material

fcab182_Supplementary_Data

## Abbreviations

bvFTD =: behavioural variant frontotemporal dementia
EB =: eigenbrain
ELISA =: enzyme-linked immunosorbent assay
FDG =: ^18^F-fluorodeoxyglucose
IWG-2 =: International Working Group-2
MMSE =: Mini-Mental Status Exam
MOANS =: Mayo Older Americans Normative Studies
MoCA =: Montreal Cognitive Assessment
NIA-AA =: National Institute on Aging—Alzheimer’s Association
STMS =: short test of mental status

## References

[1] Renner J , Burns J, Hou C, McKeel D, Storandt M, Morris J. Progressive posterior cortical dysfunction: A clinicopathologic series. Neurology. 2004;63(7):1175–1180. 15477534 10.1212/01.wnl.0000140290.80962.bf

[2] Tang-Wai DF , Graff-Radford N, Boeve BF, et al Clinical, genetic, and neuropathologic characteristics of posterior cortical atrophy. Neurology. 2004;63(7):1168–1174. 15477533 10.1212/01.wnl.0000140289.18472.15

[3] Firth NC , Primativo S, Marinescu R-V, et al Longitudinal neuroanatomical and cognitive progression of posterior cortical atrophy. Brain. 2019;142(7):2082–2095. 31219516 10.1093/brain/awz136 PMC6598737

[4] Benson DF , Davis RJ, Snyder BD. Posterior cortical atrophy. Case reports. Arch Neurol. 1988;45(7):789–793. 3390033 10.1001/archneur.1988.00520310107024

[5] Crutch SJ , Schott JM, Rabinovici GD, et al Alzheimer's Association ISTAART Atypical Alzheimer's Disease and Associated Syndromes Professional Interest Area. Consensus classification of posterior cortical atrophy. Alzheimers Dement. 2017;13(8):870–884. 28259709 10.1016/j.jalz.2017.01.014 PMC5788455

[6] Crutch SJ , Lehmann M, Schott JM, Rabinovici GD, Rossor MN, Fox NC. Posterior cortical atrophy. Lancet Neurol. 2012;11(2):170–178. 22265212 10.1016/S1474-4422(11)70289-7 PMC3740271

[7] Lehmann M , Crutch SJ, Ridgway GR, et al Cortical thickness and voxel-based morphometry in posterior cortical atrophy and typical Alzheimer's disease. Neurobiol Aging. 2011;32(8):1466–1476. 19781814 10.1016/j.neurobiolaging.2009.08.017

[8] Singh TD , Josephs KA, Machulda MM, et al Clinical, FDG and amyloid PET imaging in posterior cortical atrophy. J Neurol. 2015;262(6):1483–1492. 25862483 10.1007/s00415-015-7732-5 PMC4469094

[9] Whitwell JL , Jack CR Jr, Kantarci K, et al Imaging correlates of posterior cortical atrophy. Neurobiol Aging. 2007;28(7):1051–1061. 16797786 10.1016/j.neurobiolaging.2006.05.026 PMC2734142

[10] Vanhoutte M , Semah F, Sillaire AR, et al 18F-FDG PET hypometabolism patterns reflect clinical heterogeneity in sporadic forms of early-onset Alzheimer's disease. Neurobiol Aging. 2017;59:184–196. 28882421 10.1016/j.neurobiolaging.2017.08.009

[11] Jack CR Jr. , Wiste HJ, Weigand SD, et al Defining imaging biomarker cut points for brain aging and Alzheimer's disease. Alzheimers Dement. 2017;13(3):205–216. 27697430 10.1016/j.jalz.2016.08.005 PMC5344738

[12] Vogel JW , Young AL, Oxtoby NP, et al Four distinct trajectories of tau deposition identified in Alzheimer’s disease. Nat Med. 2021;27(5):1–11. 33927414 10.1038/s41591-021-01309-6 PMC8686688

[13] Zhang X , Mormino EC, Sun N, et al; Alzheimer’s Disease Neuroimaging Initiative. Bayesian model reveals latent atrophy factors with dissociable cognitive trajectories in Alzheimer’s disease. Proc Natl Acad Sci. 2016;113(42):E6535–E6544. 27702899 10.1073/pnas.1611073113 PMC5081632

[14] Rubin TN , Koyejo O, Gorgolewski KJ, Jones MN, Poldrack RA, Yarkoni T. Decoding brain activity using a large-scale probabilistic functional-anatomical atlas of human cognition. PLoS Comput Biol. 2017;13(10):e1005649. 29059185 10.1371/journal.pcbi.1005649 PMC5683652

[15] Groot C , Yeo BT, Vogel JW, et al Latent atrophy factors related to phenotypical variants of posterior cortical atrophy. Neurology. 2020;95(12):e1672–e1685. 32675078 10.1212/WNL.0000000000010362 PMC7713727

[16] Jack CR Jr , Holtzman DM. Biomarker modeling of Alzheimer’s disease. Neuron. 2013;80(6):1347–1358. 24360540 10.1016/j.neuron.2013.12.003 PMC3928967

[17] Gerstmann J. Syndrome of finger agnosia, disorientation for right and left, agraphia and acalculia: Local diagnostic value. Arch Neurol Psychiatry. 1940;44(2):398–408.

[18] Bálint R. Seelenlähmung des “Schauens”, optische Ataxie, räumliche Störung der Aufmerksamkeit. Eur Neurol. 1909;25:51–66.

[19] Office IL. International Standard Classification of Occupations 2008 (ISCO-08): Structure, group definitions and correspondence tables. International Labour Office; 2012.

[20] Kokmen E , Smith GE, Petersen RC, Tangalos E, Ivnik RC. The short test of mental status: Correlations with standardized psychometric testing. Arch Neurol. 1991;48(7):725–728. 1859300 10.1001/archneur.1991.00530190071018

[21] Jurica PJ , Leitten CL, Mattis S. Dementia rating Scale-2: DRS-2: Professional manual. Psychological Assessment Resources; 2001.

[22] Rey A. L’examen clinique en psychologie [The clinical examination of psychology]. Paris, France: Press Universitaire de France; 1964.

[23] Wechsler D. Wechsler Memory Scale-Revised. San Antonio: Harcourt Brace Jovanovich; 1987.

[24] Wechsler D. WMS-III: Wechsler memory scale administration and scoring manual. Psychological Corporation; 1997.

[25] Wechsler D. Scale-Revised WAI. New York: Psychological Corp.; 1981.

[26] Wechsler D. Wechsler adult intelligence scale-III. San Antonio, TX: The Psychological Corporation; 1997.

[27] Osterrieth PA. Le test de copie d'une figure complexe; contribution a l'etude de la perception et de la memoire. Arch Psychol. 1944;

[28] Reitan RM. Validity of the Trail Making Test as an indicator of organic brain damage. Percept Mot Skills. 1958;8(3):271–276.

[29] Spreen O. General intellectual ability and assessment of premorbid intelligence. A compendium of neuropsychological tests. Oxford University Press, 1998;43–135.

[30] Stroop JR. Studies of interference in serial verbal reactions. J Exp Psychol Gen. 1992;121(1):15–23.

[31] Kaplan E , Goodglass H, Weintraub S. Boston Naming Test (Experimental Version). Boston: VA Medical Center; 1976.

[32] Ruff R , Light R, Parker S, Levin H. Benton controlled oral word association test: Reliability and updated norms. Arch Clin Neuropsychol. 1996;11(4):329–338. 14588937

[33] Lucas JA , Ivnik RJ, Smith GE, et al Mayo's older Americans normative studies: Category fluency norms. J Clin Exp Neuropsychol. 1998;20(2):194–200. 9777473 10.1076/jcen.20.2.194.1173

[34] Petersen RC , Smith G, Kokmen E, Ivnik RJ, Tangalos EG. Memory function in normal aging. Neurology. 1992;42(2):396–396. 1736173 10.1212/wnl.42.2.396

[35] Ivnik RJ , Smith GE, Lucas JA, Tangalos EG, Kokmen E, Petersen RC. Free and cued selective reminding test: MOANS norms. J Clin Exp Neuropsychol. 1997;19(5):676–691. 9408798 10.1080/01688639708403753

[36] Steinberg BA , Bieliauskas LA, Smith GE, Ivnik RJ. Mayo's older Americans normative studies: Age-and IQ-adjusted norms for the trail-making test, the stroop test, and MAE controlled oral word association test. Clin Neuropsychol. 2005;19(3-4):329–377. 16120535 10.1080/13854040590945210

[37] Machulda MM , Ivnik RJ, Smith GE, et al Mayo's Older Americans Normative Studies: Visual Form Discrimination and copy trial of the Rey-Osterrieth Complex Figure. J Clin Exp Neuropsychol. 2007;29(4):377–384. 17497561 10.1080/13803390600726803

[38] Vemuri P , Whitwell JL, Kantarci K, et al Antemortem MRI based STructural Abnormality iNDex (STAND)-scores correlate with postmortem Braak neurofibrillary tangle stage. Neuroimage. 2008;42(2):559–567. 18572417 10.1016/j.neuroimage.2008.05.012 PMC3097053

[39] Senjem ML , Gunter JL, Shiung MM, Petersen RC, Jack CR Jr. Comparison of different methodological implementations of voxel-based morphometry in neurodegenerative disease. Neuroimage. 2005;26(2):600–608. 15907317 10.1016/j.neuroimage.2005.02.005 PMC2739382

[40] Jack CR Jr. , Knopman DS, Weigand SD, et al An operational approach to National Institute on Aging-Alzheimer's Association criteria for preclinical Alzheimer disease. Ann Neurol. 2012;71(6):765–775. 22488240 10.1002/ana.22628 PMC3586223

[41] Jones DT , Lowe V, Graff-Radford J, et al Patterns of neurodegeneration in dementia reflect a global functional state space. medRxiv. 2020;

[42] Turk M , Pentland A. Eigenfaces for recognition. J Cogn Neurosci. 1991;3(1):71–86. 23964806 10.1162/jocn.1991.3.1.71

[43] Yarkoni T , Poldrack RA, Nichols TE, Van Essen DC, Wager TD. Large-scale automated synthesis of human functional neuroimaging data. Nat Methods. 2011;8(8):665–670. 21706013 10.1038/nmeth.1635 PMC3146590

[44] Poldrack RA , Mumford JA, Schonberg T, Kalar D, Barman B, Yarkoni T. Discovering relations between mind, brain, and mental disorders using topic mapping. PLoS Comput Biol. 2012;8(10):e1002707. 23071428 10.1371/journal.pcbi.1002707 PMC3469446

[45] Townley RA , Graff-Radford J, Mantyh WG, et al Progressive dysexecutive syndrome due to Alzheimer’s disease: A description of 55 cases and comparison to other phenotypes. Brain Commun. 2020;2(1):fcaa068. 32671341 10.1093/braincomms/fcaa068 PMC7325839

[46] Townley RA , Syrjanen JA, Botha H, et al Comparison of the Short Test of Mental Status and the Montreal Cognitive Assessment across the cognitive spectrum. Elsevier. 2019;94(8):1516–1523. 10.1016/j.mayocp.2019.01.043 PMC6937135 31280871

[47] Scheltens NM , Tijms BM, Koene T, et al Alzheimer's Disease Neuroimaging Initiative. Cognitive subtypes of probable Alzheimer's disease robustly identified in four cohorts. Alzheimers Dement. 2017;13(11):1226–1236. 28427934 10.1016/j.jalz.2017.03.002 PMC5857387

[48] Goodale MA , Milner AD. Separate visual pathways for perception and action. Trends Neurosci. 1992;15(1):20–25. 1374953 10.1016/0166-2236(92)90344-8

[49] Kravitz DJ , Saleem KS, Baker CI, Mishkin M. A new neural framework for visuospatial processing. Nat Rev Neurosci. 2011;12(4):217–230. 21415848 10.1038/nrn3008 PMC3388718

[50] Kravitz DJ , Saleem KS, Baker CI, Ungerleider LG, Mishkin M. The ventral visual pathway: An expanded neural framework for the processing of object quality. Trends Cogn Sci. 2013;17(1):26–49. 23265839 10.1016/j.tics.2012.10.011 PMC3532569

[51] Takemura H , Rokem A, Winawer J, Yeatman JD, Wandell BA, Pestilli F. A major human white matter pathway between dorsal and ventral visual cortex. Cereb Cortex. 2016;26(5):2205–2214. 25828567 10.1093/cercor/bhv064 PMC4830295

[52] McMonagle P , Deering F, Berliner Y, Kertesz A. The cognitive profile of posterior cortical atrophy. Neurology. 2006;66(3):331–338. 16476930 10.1212/01.wnl.0000196477.78548.db

[53] Kanwisher N , McDermott J, Chun MM. The fusiform face area: A module in human extrastriate cortex specialized for face perception. J Neurosci. 1997;17(11):4302–4311. 9151747 10.1523/JNEUROSCI.17-11-04302.1997 PMC6573547

[54] Tsai PH , Teng E, Liu C, Mendez MF. Posterior cortical atrophy: Evidence for discrete syndromes of early-onset Alzheimer's disease. Am J Alzheimers Dis Other Demen. 2011;26(5):413–418. 21831859 10.1177/1533317511418955 PMC3370410

[55] Freud E , Plaut DC, Behrmann M. ‘What’ is happening in the dorsal visual pathway. Trends Cogn Sci. 2016;20(10):773–784. 27615805 10.1016/j.tics.2016.08.003

[56] Botha H , Mantyh WG, Murray ME, et al FDG-PET in tau-negative amnestic dementia resembles that of autopsy-proven hippocampal sclerosis. Brain. 2018;141(4):1201–1217. 29538658 10.1093/brain/awy049 PMC5889045

[57] Buciuc M , Botha H, Murray ME, et al Utility of FDG-PET in diagnosis of Alzheimer-related TDP-43 proteinopathy. Neurology. 2020;95(1):e23–e34. 32518145 10.1212/WNL.0000000000009722 PMC7371379

[58] Jones DT , Machulda MM, Vemuri P, et al Age-related changes in the default mode network are more advanced in Alzheimer disease. Neurology. 2011;77(16):1524–1531. 21975202 10.1212/WNL.0b013e318233b33d PMC3198977

[59] Betzel RF , Byrge L, He Y, Goñi J, Zuo X-N, Sporns O. Changes in structural and functional connectivity among resting-state networks across the human lifespan. Neuroimage. 2014;102 (Pt 2):345–357. 25109530 10.1016/j.neuroimage.2014.07.067

[60] Tsvetanov KA , Henson RN, Tyler LK, et al Cambridge Centre for Ageing and Neuroscience. Extrinsic and intrinsic brain network connectivity maintains cognition across the lifespan despite accelerated decay of regional brain activation. J Neurosci. 2016;36(11):3115–3126. 26985024 10.1523/JNEUROSCI.2733-15.2016 PMC4792930

[61] Arenaza-Urquijo EM , Przybelski SA, Lesnick TL, et al The metabolic brain signature of cognitive resilience in the 80+: Beyond Alzheimer pathologies. Brain. 2019;142(4):1134–1147. 30851100 10.1093/brain/awz037 PMC6439329

[62] Aiello Bowles EJ , Crane PK, Walker RL, et al Cognitive resilience to Alzheimer’s disease pathology in the human brain. J Alzheimers Dis. 2019;68(3):1071–1083. 30909217 10.3233/JAD-180942 PMC8357030

[63] Dickerson BC , Brickhouse M, McGinnis S, Wolk DA. Initiative AsDN. Alzheimer's disease: The influence of age on clinical heterogeneity through the human brain connectome. Alzheimers Dement. 2017;6:122–135. 10.1016/j.dadm.2016.12.007 PMC5318292 28239637

[64] Jones DT , Graff-Radford J, Lowe VJ, et al Tau, amyloid, and cascading network failure across the Alzheimer's disease spectrum. Cortex. 2017;97:143–159. 29102243 10.1016/j.cortex.2017.09.018 PMC5773067

[65] Whitwell JL , Martin P, Graff-Radford J, et al The role of age on tau PET uptake and gray matter atrophy in atypical Alzheimer's disease. Alzheimers Dement. 2019;15(5):675–685. 30853465 10.1016/j.jalz.2018.12.016 PMC6511453

[66] Josephs KA , Papenfuss SM, Duffy JR, et al Occupational differences between Alzheimer’s and aphasic dementias: Implication for teachers. Am J Alzheimers Dis Other Dement. 2013;28(6):612–616. 10.1177/1533317513494455 PMC3920458 23838322

[67] Kokmen E , Özsarfati Y, Beard CM, O'Brien PC, Rocca WA. Impact of referral bias on clinical and epidemiological studies of Alzheimer's disease. J Clin Epidemiol. 1996;49(1):79–83. 8598515 10.1016/0895-4356(95)00031-3

