# Supplementary material for: Posterior cortical atrophy phenotypic heterogeneity revealed by decoding 18F-FDG-PET
Source: Brain Commun. 2021 Aug 19;3(4):fcab182. doi: 10.1093/braincomms/fcab182 (PMC8600283; doi:10.1093/braincomms/fcab182)
Supplement: fcab182_Supplementary_Data [file fcab182_supplementary_data.docx]

Supplemental Table 1: Occupational Data

| ISCO-08 Major Group | N = 83 (%) | Examples in our cohort |
| --- | --- | --- |
| 1 - Managers | 13 (15.7%) | Executive vice president, director of operations, business owner, administrative manager |
| 2 - Professionals | 41 (49.4%) | 17 teachers, 6 health care professionals (nurse, physician), 4 accountants, 4 programmers, 2 engineers |
| 3 - Technicians and associate professionals | 4 (4.8%) | Dental hygienist, stock broker |
| 4 - Clerical support workers | 8 (9.6%) | 6 Secretary, data entry |
| 5 - Service and sales workers | 6 (7.2%) | Postal worker, Jailer, sales representative, waitress |
| 6 - Skilled agricultural, forestry and fishery workers | 2 (2.4%) | 2 Farmers |
| 7 - Craft and related trades workers | 4 (4.8%) | Iron worker, mechanic, machinist |
| 8 - Plant and machine operators and assemblers | 3 (3.6% | Bus driver, truck driver, factory assembly |
| 9 - Elementary occupations | 1 (1.2%) | Housekeeper |
| Unclassifiable | * | 6 Homemakers, 2 with unavailable information |
| * 8 patients - not included in % | | |

**Supplemental Table 2:** *Neurosynth* based eigenbrain decoding

| EB1 (-) | Negative Value | EB1 (+) | Positive Value |
| --- | --- | --- | --- |
| Visual (function) | -0.48 | Anterior cingulate (location) | 0.40 |
| Occipital lobe (location) | -0.45 | Insula (location) | 0.39 |
| Object knowledge (function) | -0.42 | Orbitofrontal (location) | 0.38 |
| Occipitotemporal (location) | -0.35 | Prefrontal cortex (location) | 0.37 |
| Perceptual language (function) | -0.32 | Pain (function) | 0.30 |
|  |  |  |  |
| EB2 (-) | Negative Value | EB2(+) | Positive Value |
| Face recognition (function) | -0.23 | Phonological (function) | 0.21 |
| Fusiform gyrus (location) | -0.20 | Verb/words (function) | 0.20 |
| Occipital lobe (location) | -0.21 | Semantic (function) | 0.19 |
| Visual (function) | -0.18 | Motor SMA (function) | 0.16 |
| Navigation/spatial (function) | -0.16 | Supplementary motor (location) | 0.16 |
|  |  |  |  |
| EB3 (-) | Negative Value | EB3 (+) | Positive Value |
| Cerebellum (location) | -0.44 | Word retrieval (function) | 0.27 |
| Motor cortex (location) | -0.23 | Comprehension (function) | 0.27 |
| Conditioning (function) | -0.24 | Semantic (function) | 0.26 |
| Movement (function) | -0.22 | Middle Temporal (location) | 0.24 |
| Target aim (function) | -0.22 | Temporoparietal | 0.23 |
|  |  |  |  |
| EB4 (-) | Negative Value | EB4 (+) | Positive Value |
| Conditioning (function) | -0.38 | Parietal (location) | 0.58 |
| Amygdala (location) | -0.38 | Fronto-parietal (location) | 0.40 |
| Hippocampus (location) | -0.36 | Attention (function) | 0.39 |
| Memory (function) | -0.34 | Premotor cortex (location) | 0.38 |
| Orbitofrontal (location) | -0.32 | Execution (function) | 0.37 |
|  |  |  |  |
| EB5 (-) | Negative Value | EB5 (+) | Positive Value |
| Occipital lobe (location) | -0.43 | Insula (location) | 0.22 |
| Cerebellar (location) | -0.42 | Anterior cingulate cortex | 0.22 |
| Primary visual (location) | -0.37 | Prefrontal cortex (location) | 0.21 |
| Lingual gyrus (location) | -0.29 | Amygdala | 0.16 |
| Occipitotemporal (location) | -0.25 | Pain | 0.16 |
|  |  |  |  |
| EB6 (-) | Negative Value | EB6 (+) | Positive Value |
| Motor cortex (location) | -0.35 | Semantic (function) | 0.36 |
| Movement (function) | -0.347 | Comprehension (function) | 0.30 |
| Premotor cortex (location) | -0.32 | Middle temporal (location) | 0.30 |
| Motor imagery (function) | -0.30 | Theory of mind (function) | 0.30 |
| Hand (function/location) | -0.30 | Prefrontal cortex (location) | 0.27 |
|  |  |  |  |
| EB7 (-) | Negative Value | EB7 (+) | Positive Value |
| Cerebellum (location) | -0.42 | Face recognition (function) | 0.25 |
| Motor cortex (location) | -0.34 | Fusiform gyrus (location) | 0.24 |
| Movement (function) | -0.34 | Prefrontal (location) | 0.24 |
| Sensory cortex (location) | -0.31 | Social awareness (function) | 0.22 |
| Premotor cortex (location) | -0.30 | Anterior temporal (location) | 0.22 |
|  |  |  |  |
| EB8 (-) | Negative Value | EB8 (+) | Positive Value |
| Somatosensory (location) | -0.17 | Primary visual (location) | 0.17 |
| Insula cortex (location) | -0.15 | Lingual gyrus (location) | 0.12 |
| Hippocampus (location) | -0.14 | Medial frontal (location) | 0.11 |
| Posterior insula (location) | -0.14 | Intention (function) | 0.11 |
| Medial temporal (location) | -0.13 | Motion detection (function) | 0.10 |


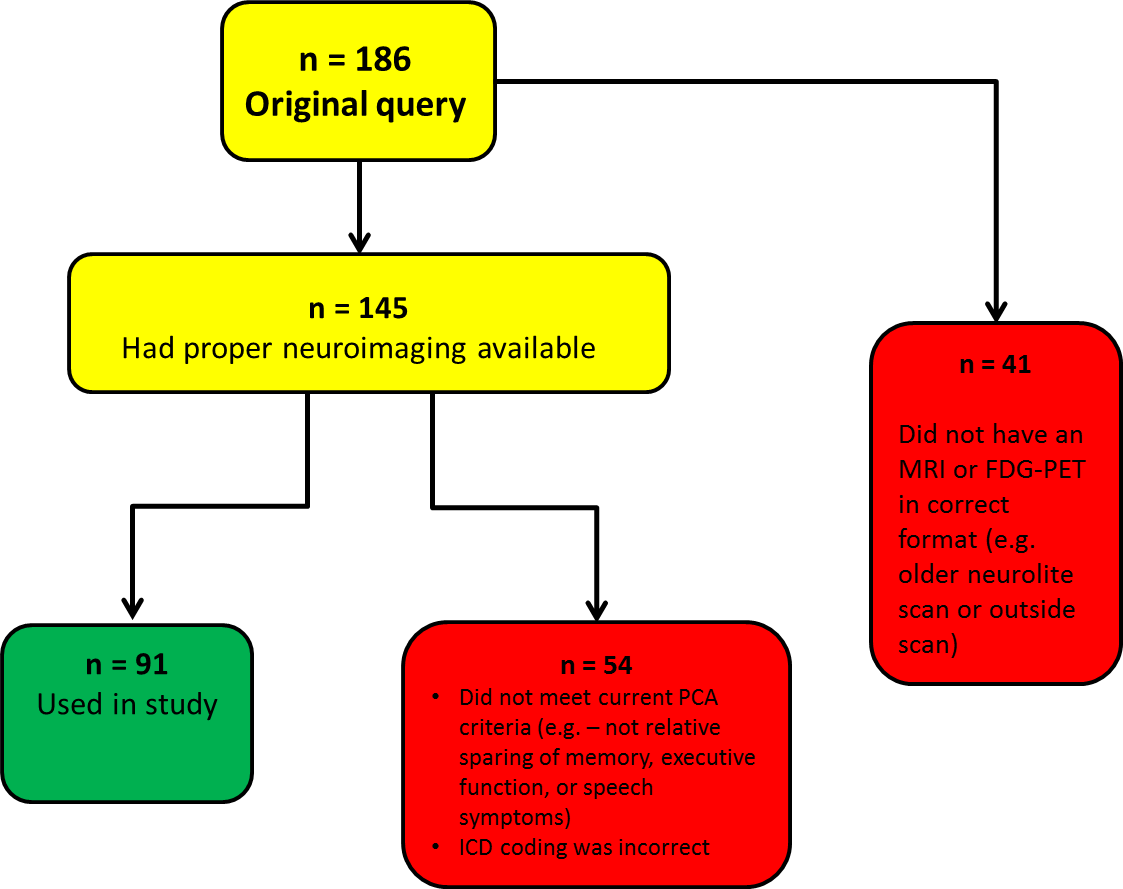


Supplemental Fig. 1: Flow diagram for participant selection in the study
